# Dynamic Antibody Response and Hybrid Immunity Following Multiple COVID-19 Vaccine Doses and Infection: A Case Study

**DOI:** 10.7759/cureus.45531

**Published:** 2023-09-19

**Authors:** Sitthichai Kanokudom, Nungruthai Suntronwong, Thaneeya Duangchinda, Nasamon Wanlapakorn, Yong Poovorawan

**Affiliations:** 1 Center of Excellence in Clinical Virology, Faculty of Medicine, Chulalongkorn University, Bangkok, THA; 2 Molecular Biology of Dengue and Flaviviruses Research Team, National Center for Genetic Engineering and Biotechnology (BIOTEC) National Science and Technology Development Agency, Pathum Thani, THA; 3 Center of Excellence In Clinical Virology, Faculty of Medicine, Chulalongkorn University, Bangkok, THA

**Keywords:** breakthrough infection, neutralization, omicron, receptor binding domain (rbd), covid-19 vaccine, sars-cov-2

## Abstract

This case study highlights the dynamic nature of the antibody response to SARS-CoV-2 in a vulnerable subject aged 70 years between 2021 and 2023. This individual had been vaccinated with six doses of the ancestral (Wuhan-Hu-1) COVID-19 vaccine and had a breakthrough infection 126 days after receiving Covovax™­ (CO) as the sixth dose. The serostatus for total immunoglobulin specific to the receptor binding domain (total RBD Ig) changed from negative to positive following a two-dose CoronaVac (CV) vaccination, indicating a successful immune response. Booster doses, including AZD1222 (AZ), half-dose BNT162b2 (PF), and CO, increased the total RBD Ig levels, except for CV. The individual experienced a breakthrough infection by the Omicron BA.5 variant, leading to a substantial surge in total RBD Ig to over 10^5^ U/mL. This generated sustained and extended antibody persistence, with the half-life of total RBD Ig lasting approximately 103.6 days. Furthermore, it has been observed that this breakthrough infection generated the highest neutralizing antibodies against BA.5, followed by XBB.1.5, BQ.1.1, and BA.2.75, respectively.

## Introduction

SARS-CoV-2 was initially identified in China in late December 2019, leading to the COVID-19 pandemic. A year later, vaccination campaigns were approved for emergency use and implemented worldwide. The previous study showed that the IgG antibody response peaked around two to four weeks after a second dose of vaccination and declined thereafter [1]. A third booster vaccination could rapidly induce elevated levels of IgG antibody, which consequently waned at a slower rate compared to the response observed after a second dose vaccination [2]. Additionally, the vaccine effectiveness against infection following a third dose gradually declined over time, and this effectiveness could be restored by the administration of a fourth dose [3]. Nevertheless, a real-world cross-sectional study involving a total of 4126 individuals who received two to four doses demonstrated that antibody production and its durability correlated with a higher number of vaccine doses [4].

Despite global vaccination efforts, there was inadequate protection against the SARS-CoV-2 variants, resulting in a large number of patients susceptible to COVID-19 infection [5-6]. Currently, the Omicron variants have become globally dominant. The Omicron variant possesses a high number of mutations, particularly in the spike protein, including the receptor binding domain (RBD). This leads to a notable antigenic distance from the original strains [7]. The previous study suggested that the Omicron variant exhibited resistance to neutralization in serum samples from individuals who had been infected with pre-Omicron variants as well as those who had received a three-dose vaccine regimen [7-8]. However, there has been no good study of the dynamic antibody response and neutralization against Omicron variants in individuals with multiple booster doses and breakthrough infections.

In this study, a patient expressed their willingness to monitor their blood tests since the COVID-19 outbreak. As it was practically not feasible to obtain blood samples like this in almost any setting, we obtained consent and conducted the study to monitor their antibody levels in response to SARS-CoV-2. The study aimed to evaluate the dynamics of antibody responses to SARS-CoV-2 in an individual who received six doses of the ancestral COVID-19 vaccine and experienced a breakthrough infection. Additionally, we assessed the neutralizing profiles against Omicron variants BA.5, BA.2.75, BQ.1.1, and XBB.1.5.

## Case presentation

**Figure 1:**
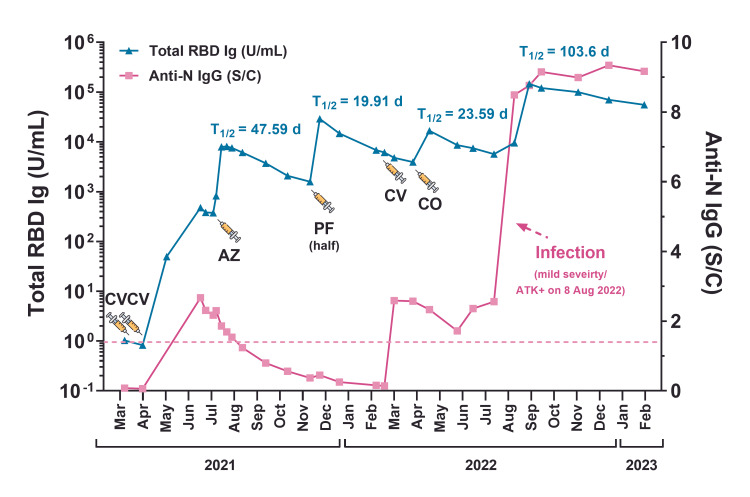
A prolonged exploration of antibody responses to SARS-CoV-2 in a 70-year-old individual following exposure to both COVID-19 vaccination and SARS-CoV-2 infection. Total immunoglobulin specific to receptor binding domain (total RBD Ig) (U/mL) (left Y-axis) and IgG specific to nucleoprotein (anti-N IgG) (S/C) (right Y-axis) of the individual were tracked between March 2021 and February 2023. The syringe icons indicate the time points of vaccination. The dashed line indicated seronegativity of anti-N IgG (<1.4 S/C). Abbreviation: AZ: AZD1222; CO: Covovax™; CV: CoronaVac; PF: BNT162b2; ATK: antigen test kit; d: day; T1/2: half-life of total RBD Ig (day).

The patient is a healthy 70-year-old Thai male physician who has been working on the frontlines during the COVID-19 outbreak. This patient had personal health concerns and wanted to know their antibody levels’ status throughout their period of work. He voluntarily underwent blood tests for the antibody response to SARS-CoV-2 antigens, which included total immunoglobulin specific to the receptor binding domain (total RBD Ig) and IgG specific to nucleoprotein (anti-N IgG). Blood samples were collected at 30 time points between March 16, 2021, and February 6, 2023, and subsequently assigned to blood testing (Figure 1). Initially, his serostatus for total RBD Ig and anti-N IgG was negative, indicating that he had not previously been exposed to SARS-CoV-2 and had never received any COVID-19 vaccine. After receiving the two doses of CoronaVac (CV), his serostatus changed to positive (Figure 1, blue line).

Furthermore, he received several booster doses of the COVID-19 vaccine, including AZD1222 (AZ), half-dose BNT162b2 (PF), CV, and Covovax™ (CO), respectively. The total RBD Ig levels increased after administration of the booster dose (except for CV). Nevertheless, the total RBD Ig exhibited a gradual decline over time, with calculated half-lives (T1/2) demonstrated in Figure 1 using a one-phase decay model. This person experienced a breakthrough infection by SARS-CoV-2, leading to a positive result on an antigen test kit (ATK+) 126 days following the sixth dose on August 8, 2022. The date of infection coincided with the predominant phase of the SARS-CoV-2 Omicron sublineage BA.5. During the infection, he experienced a fever of 37.5 °C for one day and a mild cough for five days. He received monulpiravir 800 mg twice a day for five days. ATK became negative on day 6 after the onset of symptoms. Notably, the total RBD Ig exhibited a remarkable 25-fold surge, rising from 5,691 U/mL before the breakthrough infection to 146,528 U/mL by day 29 subsequent to the ATK+ test. (Figure 1). Additionally, the total RBD Ig level showed a gradual decrease over time, with a calculated T1/2 of 103.6 days. The peak level of total RBD Ig was noted at 29 days following the breakthrough infection in comparison with all 30 testing time points.

In parallel, the sera samples were examined for the presence of anti-N IgG. The findings revealed that the person exhibited a positive test result for anti-N IgG after receiving the inactivated, whole-virion COVID-19 vaccine (CoronaVac) as booster doses (the second and fifth doses). The rise in anti-N IgG was also observed following the COVID-19 breakthrough infection (Figure 1, pink line).

The sera samples collected before and after the administration of booster doses (3rd-6th doses), as well as samples collected after the breakthrough infection, were evaluated for their neutralizing activity using the focus reduction neutralization test (FRNT50) against live viruses, including Omicron BA.5, BA.2.75, BQ.1.1, and XBB.1.5, respectively (with the following GISAID accession numbers: EPI_ISL_17646230, EPI_ISL_1764623, EPI_ISL_17646271, and EPI_ISL_17646336, respectively). The administration of the third dose of AZ did not result in the detection of neutralizing activity against the Omicron variants. However, following the administration of the fourth half-dose PF and the sixth CO booster vaccination, this individual elicited neutralizing antibody responses against the Omicron BA.5, BA.2.75, and XBB.1.5 variants (Table 1). Furthermore, it was found that hybrid immunity enhanced neutralizing activities against the Omicron BA.5, BA.2.75, BQ.1.1, and XBB.1.5 variants compared to vaccination alone. Among these variants, the highest level of neutralization was observed against BA.5, followed by XBB.1.5, BQ.1.1, and BA.2.75, respectively (Table 1). This data suggests that this individual might have been infected with Omicron BA.5.

**Table 1:** Neutralizing antibody titer after exposure with vaccine and infection. Abbreviation: AZ: AZD1222; CO: Covovax^TM^; CV: CoronaVac; PF: BNT162b2; ATK: antigen test kit; Pre: pre-immunization; Post: post-immunization; dbi.: day before infection; dpi.: day post-infection; Total RBD Ig: total immunoglobulin specific to receptor binding domain; FRNT: focus reduction neutralization test.

Antigen exposure status		Total RBD Ig (U/ml)	FRNT_50_ (reciprocal serum dilution)			
			BA.5	BA.2.75	BQ.1.1	XBB.1.5
Third dose (AZ)	Pre	376.1	<20	<20	<20	<20
	Post 18 days	8,095	<20	<20	<20	<20
	Post 25 days	7,597	<20	<20	<20	<20
Fourth dose (PF)	Pre	1,592	<20	28	<20	20
	Post 13 days	29,150	169	26	<20	48
Fifth dose (CV)	Pre	6,145	29	20	<20	20
	Post 13 days	4,805	<20	25	<20	25
Sixth dose (CO)	Pre	3,940	<20	<20	<20	<20
	Post 22 days	16,678	350	30	20	22
ATK+	18 dbi.	5,691	66	25	<20	<20
	29 dpi.	146,528	7,693	553	1,857	1,974

## Discussion

This physician's duties are involved in COVID-19. His regular blood tests have been conducted over a period of two years. Nevertheless, obtaining long-term serum samples with multiple time points has posed a challenge. We believe that the knowledge gained in this study could be beneficial for healthcare professionals or the general public, who may not have access to blood tests. This study demonstrated the dynamics of antibody responses to SARS-CoV-2 antigens in an individual over a span of nearly two years. His total RBD Ig serostatus changed from negative to positive after receiving the two doses of CV. The administration of multiple booster doses, such as AZ, half-dose PF, and CO, further enhanced total RBD Ig levels, except for the CV vaccine. Individuals at risk for severe COVID-19 are recommended to receive the booster dose, as vaccine-induced immunity wanes over time. Previous studies indicated a positive correlation between antibody levels and vaccine efficacy [9]. Furthermore, administration of the fourth dose further increased the vaccine's effectiveness against severe diseases compared to receiving three doses [10]. Nevertheless, these findings suggested that the inactivated, whole-virion COVID-19 vaccine as a booster dose did not increase the total RBD Ig compared to other types of COVID-19 vaccine platforms. This finding was supported by the evidence that inactivation of SARS-CoV-2 using beta-propiolactone diminished antigenic potentials [11-12]. Additionally, these results were in alignment with recent studies demonstrating that the incorporation of inactivated whole-virion vaccines like CV or BBIBP-CorV as booster doses resulted in lower levels of RBD-specific antibodies compared to other platforms of COVID-19 vaccines [13-14].

The half-lives were calculated based on the gradual decline in total RBD Ig levels for each dose. Our data suggested that the estimated half-life after the booster dose with the third dose of viral vector vaccine (AZ) was longer compared to both the fourth dose of mRNA vaccine (PF) and the sixth dose of subunit vaccine (CO). Our findings were consistent with the COV-BOOST trial, indicating that the administration of adenoviral vector vaccines (AZ and Ad26) after the PF/PF prime led to the highest persistence of anti-spike IgG up to 84 days when compared to the administration of mRNA and subunit vaccines [15]. However, the comparison of half-lives in total RBD Ig across vaccine doses (3rd, 4th, and 6th) in this individual remained unclear. This finding was similar to the previous study, where the calculated half-lives of binding antibody titers were 66 days for those who received a three-dose mRNA vaccine regimen and 40 days for those on a four-dose regimen [16].

Our finding provided evidence that despite an individual receiving multiple ancestral strain-based vaccines, breakthrough infections have occurred, especially in this case coinciding with the Omicron BA.5. To date, there is no established threshold for protective antibody levels against SARS-CoV-2. The results demonstrated that hybrid immunity elicited strong immune responses and maintained sustained antibody levels in contrast to vaccination alone. Furthermore, the present study revealed that in the serum sample of an individual following breakthrough infection, BA.5 was effectively neutralized, and broad reactivity was observed against XBB.1.5, BQ.1.1, and BA.2.75. This was supported by imprint immunity, which suggested that hybrid immunity-induced memory B cells generate cross-reactive antibodies against various Omicron sublineages [17]. In agreement with this, Cao et al. demonstrated that postvaccination Omicron BA.1 infection recalled humoral immune memory against ancestral spike protein [18]. Furthermore, this infection enhanced BA.1 neutralization and cross-neutralization of Omicron sublineages compared with the non-infected three-dose ancestral vaccination group [18].

## Conclusions

This study has provided evidence that vaccination stimulated antibody responses, but the antibody levels gradually declined over time. To sustain a high level of immunity and protection against severe disease, administering booster doses was recommended. This study emphasized that breakthrough infection increased antibody levels and neutralizing activity against SARS-CoV-2 variants. The COVID-19 vaccination will be similar to the seasonal influenza vaccination campaigns, with the selection of vaccine strains relying on forecasting the predominant strains for the following year. Vaccination will primarily target high-risk groups to mitigate the severity of the disease.

## References

[1] Shrotri M, Fragaszy E, Nguyen V (2022). Spike-antibody responses to COVID-19 vaccination by demographic and clinical factors in a prospective community cohort study. Nat Commun.

[2] Gilboa M, Regev-Yochay G, Mandelboim M (2022). Durability of immune response after COVID-19 booster vaccination and association with COVID-19 Omicron infection. JAMA Netw Open.

[3] Regev-Yochay G, Gonen T, Gilboa M (2022). Efficacy of a fourth dose of Covid-19 mRNA vaccine against Omicron. N Engl J Med.

[4] Yorsaeng R, Atsawawaranunt K, Suntronwong N (2023). SARS-CoV-2 antibody dynamics after COVID-19 vaccination and infection: a real-world cross-sectional analysis. Vaccines (Basel).

[5] Yamamoto S, Matsuda K, Maeda K (2023). Omicron BA.1 neutralizing antibody response following Delta breakthrough infection compared with booster vaccination of BNT162b2. BMC Infect Dis.

[6] Tan ST, Kwan AT, Rodríguez-Barraquer I (2023). Infectiousness of SARS-CoV-2 breakthrough infections and reinfections during the Omicron wave. Nat Med.

[7] Hoffmann M, Krüger N, Schulz S (2022). The Omicron variant is highly resistant against antibody-mediated neutralization: Implications for control of the COVID-19 pandemic. Cell.

[8] Pulliam JR, van Schalkwyk C, Govender N (2022). Increased risk of SARS-CoV-2 reinfection associated with emergence of Omicron in South Africa. Science.

[9] Feng S, Phillips DJ, White T (2021). Correlates of protection against symptomatic and asymptomatic SARS-CoV-2 infection. Nat Med.

[10] Magen O, Waxman JG, Makov-Assif M (2022). Fourth Dose of BNT162b2 mRNA Covid-19 Vaccine in a Nationwide Setting. N Engl J Med.

[11] Gupta D, Parthasarathy H, Sah V, Tandel D, Vedagiri D, Reddy S, Harshan KH (2021). Inactivation of SARS-CoV-2 by β-propiolactone causes aggregation of viral particles and loss of antigenic potential. Virus Res.

[12] Heinz FX, Stiasny K (2021). Distinguishing features of current COVID-19 vaccines: knowns and unknowns of antigen presentation and modes of action. NPJ Vaccines.

[13] Wanlapakorn N, Suntronwong N, Phowatthanasathian H (2022). Safety and immunogenicity of heterologous and homologous inactivated and adenoviral-vectored COVID-19 vaccine regimens in healthy adults: a prospective cohort study. Hum Vaccin Immunother.

[14] Kanokudom S, Assawakosri S, Suntronwong N (2022). Safety and immunogenicity of the third booster dose with inactivated, viral vector, and mRNA COVID-19 vaccines in fully immunized healthy adults with inactivated vaccine. Vaccines (Basel).

[15] Liu X, Munro AP, Feng S (2022). Persistence of immunogenicity after seven COVID-19 vaccines given as third dose boosters following two doses of ChAdOx1 nCov-19 or BNT162b2 in the UK: Three month analyses of the COV-BOOST trial. J Infect.

[16] Arunachalam PS, Lai L, Samaha H (2023). Durability of immune responses to mRNA booster vaccination against COVID-19. J Clin Invest.

[17] Park YJ, Pinto D, Walls AC (2022). Imprinted antibody responses against SARS-CoV-2 Omicron sublineages. Science.

[18] Cao Y, Yisimayi A, Jian F (2022). BA.2.12.1, BA.4 and BA.5 escape antibodies elicited by Omicron infection. Nature.

